# Effects of Whole-Body Cryostimulation on Stress Biomarkers and Psychological Well-Being in Parkinson’s Disease: A Pilot Study

**DOI:** 10.3390/jcm15041602

**Published:** 2026-02-19

**Authors:** Paolo Piterà, Stefania Cattaldo, Riccardo Cremascoli, Laura Bianchi, Elisa Prina, Federica Verme, Erica Sabattini, Lorenzo Priano, Alessandro Mauro, Paolo Capodaglio

**Affiliations:** 1 Department of Neurosciences “Rita Levi Montalcini”, University of Turin, 10126 Turin, Italy; e.prina@auxologico.it (E.P.); lorenzo.priano@unito.it (L.P.); alessandro.mauro@unito.it (A.M.); 2 Laboratory of Clinical Neurobiology, IRCCS Istituto Auxologico Italiano, San Giuseppe Hospital, 28824 Verbania, Italy; r.cremascoli@auxologico.it (R.C.); l.bianchi@auxologico.it (L.B.); e.sabattini@auxologico.it (E.S.); 3 Research Laboratory in Biomechanics, Rehabilitation and Ergonomics, IRCCS Istituto Auxologico Italiano, San Giuseppe Hospital, 28824 Verbania, Italy; f.verme@auxologico.it (F.V.); p.capodaglio@auxologico.it (P.C.); 4 Department of Biomedical, Surgical and Dental Sciences, University of Milan, 20122 Milan, Italy

**Keywords:** Parkinson’s disease, rehabilitation, whole-body cryostimulation, PD non-motor symptoms, non-pharmacological treatment in PD, neuroendocrine modulation, stress response, psychological well-being

## Abstract

**Background**: Parkinson’s disease (PD) is a progressive neurodegenerative disorder characterized not only by motor impairments but also by debilitating non-motor symptoms (NMS) such as anxiety, depression, fatigue, and sleep disturbances. These symptoms are often resistant to dopaminergic therapies and significantly impact patients’ quality of life. Whole-body cryostimulation (WBC) has emerged as a promising non-pharmacological intervention with potential effects on stress modulation and psychological well-being. **Materials and Methods**: In total, 14 patients with idiopathic PD underwent 10 WBC sessions (−110 °C for 2 min) over two weeks. Blood samples for cortisol and serotonin were collected before and after the first and last sessions. Patients completed standardized questionnaires evaluating anxiety (STAI-Y1, Y2), depression (BDI), fatigue (FSS), sleep quality (SCI), and daytime sleepiness (ESS) at baseline and after the final session. **Results**: Significant reductions in serum cortisol and improvements in serotonin levels were observed. Psychological assessments showed decreased anxiety and depression scores, with improvements in fatigue and sleepiness (*p* < 0.05 for most scales). **Discussion and Conclusions**: Repeated WBC sessions were safe and well-tolerated and were associated with biochemical and psychological improvements. These preliminary findings support WBC as a promising complementary intervention for alleviating NMS in PD. Further randomized controlled studies are warranted to confirm these results.

## 1. Introduction

Parkinson’s disease (PD) is a multifaceted and progressively disabling neurodegenerative disorder, primarily associated with the loss of dopaminergic neurons in the substantia nigra and widespread Lewy body pathology [1]. While cardinal motor manifestations such as tremor, rigidity, gait impairments and bradykinesia are well recognized, increasing attention is being directed towards the broad spectrum of non-motor symptoms (NMS), which can emerge early in the disease course and frequently persist throughout its progression [2]. These include neuropsychiatric disturbances (e.g., depression, anxiety) [3], fatigue, sleep disorders, pain, cognitive dysfunction, and cardiovascular autonomic dysregulation [4], all of which significantly affect patients’ quality of life and global functioning [5]. Among these, depression and anxiety are the most prevalent non-motor symptoms, affecting approximately 30–50% of individuals with PD [3]. Fatigue and sleep disturbances, often underdiagnosed, can affect up to half of patients and may precede motor symptoms by several years. Importantly, these psychological and somatic symptoms often have a greater impact on perceived health status than motor deficits themselves, as they undermine motivation, treatment adherence, and engagement in rehabilitation programs [6]. Sleep disturbances further intensify fatigue and emotional distress, triggering a vicious cycle that impairs physical, cognitive, and social participation [7].

From a pathophysiological perspective, Parkinson’s disease is increasingly recognized as a multisystem disorder involving not only dopaminergic neurodegeneration but also inflammatory and metabolic mechanisms. Chronic neuroinflammation, driven by microglial activation and pro-inflammatory cytokines, together with metabolic dysregulation such as hypoglycemic episodes and increased glycemic variability, may contribute to neuronal stress and disease progression [8]. These interacting pathways are thought to play a role in the heterogeneity of Parkinson’s disease, particularly in the development and persistence of non-motor symptoms including fatigue, mood disturbances, and autonomic dysfunction [8,9].

Despite their clinical relevance, NMS of PD remains insufficiently addressed by dopaminergic therapies, which mainly target motor deficits [10]. Pharmacological treatments for mood and sleep disorders—such as antidepressants, anxiolytics, or sedative agents—often show limited efficacy and may be poorly tolerated in elderly or frail individuals with PD, due to polypharmacy, altered pharmacokinetics, or side effects [11,12]. This therapeutic gap underscores the need for adjunctive non-pharmacological interventions that are safe, accessible, and capable of modulating both psychological symptoms and underlying neurobiological mechanisms [13].

In this context, interventions capable of influencing neuroendocrine stress responses and autonomic function, such as structured physical activity, thermal therapies, or other rehabilitative strategies, have gained growing interest. Modulation of the hypothalamic–pituitary–adrenal (HPA) axis, neuroinflammation, and serotonergic tone may represent promising avenues to relieve mood symptoms and fatigue in PD [14]. Exploring such integrative approaches is essential to developing multimodal rehabilitation strategies that address the full clinical burden of PD and promote sustainable improvements in well-being.

Whole-body cryostimulation (WBC), also referred to as whole-body cryotherapy, is a physical therapy which involves brief exposure of the entire body to extreme cold (typically around −110 °C to −140 °C for 2–3 min) in a temperature-controlled chamber [15]. Originally developed and popularized in sports medicine for enhancing recovery and performance, WBC has more recently gained attention in clinical medicine for its broad potential health benefits [16,17].

The extreme cold stimulus triggers a cascade of physiological responses: skin temperature receptors send afferent signals that elicit reflex cutaneous vasoconstriction and a transient central blood volume shift, activating baroreceptors. This response is thought to “train” the autonomic nervous system by initially stimulating sympathetic outflow, followed by a rebound increase in parasympathetic tone once the body re-warms. Improvements in heart rate variability and parasympathetic indices have been observed after WBC exposures [18,19].

Because PD patients often have reduced parasympathetic cardiac control, WBC’s autonomic effects may offer valuable therapeutic benefits. In PD, repeated WBC sessions have been shown to reduce baseline sympathetic activity and shift cardiac autonomic balance toward parasympathetic dominance in healthy individuals [20].

Beyond autonomic modulation, WBC may exert several beneficial systemic effects that could alleviate PD’s non-motor symptoms. Cold exposure induces a hormetic stress response, resulting in the release of various neuroendocrine factors. For example, studies in healthy adults have documented that WBC can impact circulating cortisol, while elevating levels of endorphins and anti-inflammatory cytokines [21,22,23].

There is also evidence that cryotherapy can influence neurotransmitters associated with mood and fatigue: in patients undergoing WBC as an adjunct to exercise therapy, significant increases in plasma serotonin and melatonin concentrations have been observed post-treatment [24]. These biochemical changes provide a plausible basis for the mood-elevating and sleep-improving effects anecdotally reported with cryostimulation. In clinical studies outside of PD, WBC has shown promise in ameliorating depressive and anxiety symptoms when used as an add-on therapy [25,26].

Moreover, WBC in combination with standard rehabilitation has demonstrated beneficial effects across various neurological conditions, including phantom limb syndrome (PLP) [27], cerebral palsy-related spasticity [28] and for tinnitus-related disorders [29], supporting its potential as a complementary strategy in neurorehabilitation settings.

Considering the constellation of non-motor symptoms in PD and the mechanistic rationale for cryostimulation, we hypothesized that WBC could serve as a valuable adjunctive therapy in PD rehabilitation. By acutely lowering stress hormone levels (cortisol) and boosting neurotransmitters linked to mood and vigor (such as noradrenaline and serotonin), WBC may help break the cycle of fatigue, depression, and poor sleep that plagues many PD patients. Enhanced parasympathetic activity from repeated cold exposure could also potentially improve autonomic stability, which is relevant given the prevalence of orthostatic blood pressure issues and other autonomic dysfunctions in PD [20].

To explore these possibilities, we conducted a pilot study in which 14 patients with PD underwent a regimen of 10 whole-body cryostimulation sessions (−110 °C for 2 min each, over two weeks). We collected objective biomarkers of stress and well-being including plasmatic cortisol and serotonin levels pre- and post-intervention, alongside a battery of validated questionnaires evaluating daytime sleepiness (Epworth Sleepiness Scale), fatigue (Fatigue Severity Scale, FSS), anxiety (State-Trait Anxiety Inventory, STAI forms I and II), depression (Beck Depression Inventory, BDI), and sleep quality/insomnia (Sleep Condition Indicator, SCI).

The overarching aim was to assess whether repeated WBC can safely improve the general health status of PD patients, reflected in better mood, energy, and sleep, thereby enhancing their overall quality of life and complementing conventional motor rehabilitation. In this article, we present the rationale and initial findings of this pilot trial, which suggest that whole-body cryostimulation may indeed confer broad therapeutic benefits for individuals affected by Parkinson’s disease.

## 2. Materials and Methods

### 2.1. Participants

Between June 2021 and March 2024, adult inpatients with Parkinson’s disease admitted to the Neurologic Unit of San Giuseppe Hospital, IRCCS Istituto Auxologico Italiano, Piancavallo (VB), agreed to participate in this study. Patients were given full information about the scope and methodology of the study, which was conducted in conformity with the Declaration of Helsinki of the World Medical Association and approved by the Ethics Committee of the Istituto Auxologico Italiano (reference: 2021_05_18_14). Written and verbal informed consent was obtained from all patients. The sample analyzed in this study represents a subset of a larger cohort originally collected to investigate broader trends in the impact of WBC in patients with metabolic or neurological disease, fibromyalgia or healthy normal weight/overweight patients (study registration: NCT05443100). The subgroup selected for this specific analysis was chosen based on the presence of a diagnosis of Parkinson’s disease

### 2.2. Study Design

**Figure 1:**
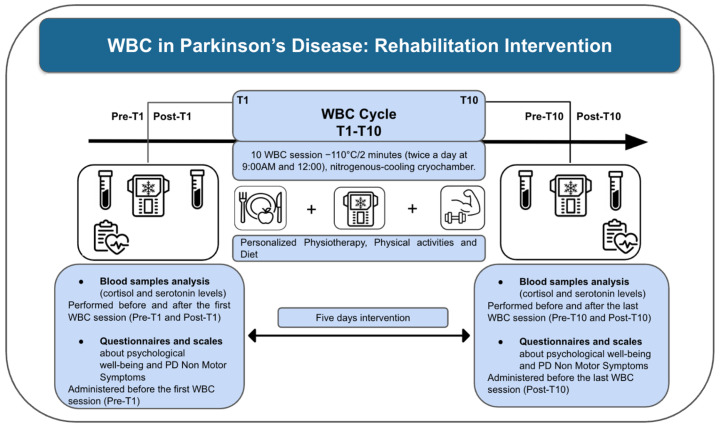
Study design.

This study aimed to investigate the effects of whole-body cryostimulation (WBC), included in a multidisciplinary rehabilitation program, on psychological well-being and general health in patients with Parkinson’s disease (PD). The stages of the study protocol are summarized in Figure 1 and include patient enrollment, intervention, and data analysis. Patients were consecutively recruited from a comprehensive, multidisciplinary rehabilitation program for PD, which incorporated a cycle of 10 WBC sessions performed in a medical nitrogen-cooled cryochamber (Arctic, CryoScience, Rome, Italy). Before starting the intervention, all participants underwent a one-minute familiarization session at −110 °C. Subsequently, they received 10 WBC treatments, each lasting two minutes, administered over five consecutive days.

Blood samples were collected immediately before and after the first (Pre-T1 and post-T10) and immediately before and after the last (Pre-T10 and Post-T10) WBC session to assess circulating cortisol and serotonin levels. In addition, participants completed a battery of validated questionnaires assessing mood, fatigue, sleep, and anxiety—specifically, the Beck Depression Inventory (BDI), State-Trait Anxiety Inventory (STAI forms I and II), Epworth Sleepiness Scale, Fatigue Severity Scale (FSS), and Sleep Condition Indicator (SCI)—administered prior to the first WBC session and after completion of the final session.

### 2.3. Participant Eligibility

Participants were considered eligible for inclusion if they had a confirmed diagnosis of idiopathic Parkinson’s disease (PD) without the presence of motor fluctuations (specifically, no episodes of motor “OFF” phases) and without signs of cognitive impairment, as indicated by a Mini-Mental State Examination (MMSE) score of at least 24 [30].

Additional inclusion criteria required the ability to maintain an upright standing position independently for a minimum of 3 min, as well as a stable pharmacological regimen, with no modifications in their daily levodopa dosage throughout the cryostimulation protocol. Therefore, all enrolled patients were cognitively preserved, with no evidence of dementia or clinically relevant cognitive impairment at baseline, and were able to fully understand and comply with the study procedures and assessments.

Only patients with idiopathic PD were recruited, with a clearly defined bradykinetic-rigid clinical subtype, in order to ensure clinical homogeneity of the sample.

The administered levodopa equivalent daily dose (LEDD)—which accounts for the cumulative effect of all antiparkinsonian medications, including levodopa and dopamine agonists—ranged between 300 and 1250 mg. Participants were under stable dopaminergic treatment and did not undergo any pharmacological adjustments during the intervention period, thus minimizing potential confounding effects related to medication changes. Recruitment was limited to patients diagnosed with the bradykinetic-rigid form of idiopathic PD, presenting with mild to moderate disease severity, classified between stages 1 and 3 on the Hoehn and Yahr scale.

According to the 2025 position paper from the WBC Working Group of the International Institute of Refrigeration on WBC’s contraindications [15] patients were excluded from participation if they presented with severe psychiatric disorders, active cancer or recent oncologic history, acute cardiovascular or respiratory conditions, unstable blood pressure, intolerance to cold, asthma, claustrophobia, recent changes in their pharmacotherapy, cryoglobulinemia, unexplained weight loss in the preceding three months, or body temperature exceeding 37.5 °C.

Moreover, a specific pharmacological screening was performed to exclude individuals taking monoamine oxidase inhibitors (MAOIs) or antipsychotic medications, given their potential influence on neurochemical and neuroendocrine outcomes, to avoid potential interference with neurochemical assessments. To maintain sample homogeneity and reduce the risk of confounding factors, patients with atypical Parkinsonian syndromes, such as multiple system atrophy (MSA) or progressive supranuclear palsy (PSP), were not considered eligible.

All participants underwent a detailed clinical assessment by an experienced neurologist (R.C.) before the start of the study to confirm compliance with the inclusion criteria and to rule out any contraindications to WBC. This assessment included confirmation of disease subtype, disease stage, clinical stability, and ongoing pharmacological treatment. Only those meeting all safety and eligibility requirements were enrolled in the trial.

### 2.4. Multidisciplinary Rehabilitation Program

All participants were admitted to an intensive, multidisciplinary rehabilitation program with an average duration of seven days. This comprehensive intervention included individualized nutritional counselling, psychological support, a standardized protocol of 10 WBC sessions, and structured physical activity, all performed under close medical supervision during the hospitalization period.

Following an initial nutritional evaluation, patients were prescribed a personalized Mediterranean-style diet, calibrated to provide between 1300 and 1500 kilocalories per day. The dietary composition was carefully balanced, consisting of approximately 70 to 74 g of protein (representing 20–21% of total energy intake), 42 to 47 g of fat (accounting for 29–30% of total daily energy, with saturated fats restricted to below 8%), and 162 to 190 g of carbohydrates (covering about 50–51% of daily caloric intake, with simple sugars limited to less than 15%). Fiber intake was standardized at 30 g per day, primarily derived from fresh vegetables.

Additionally, each patient followed an individualized physiotherapy plan, adapted to their clinical condition, stage of Parkinson’s disease, and functional abilities. The rehabilitation sessions lasted 60 min per day and incorporated a progressive aerobic component, exercises targeting postural stability and balance, as well as muscle-strengthening activities. All sessions were conducted under the direct supervision of a physiotherapist, with continuous monitoring of exertion levels. Physical activity was immediately paused if the participant reported a perceived exertion score of 5 or higher on the Borg Rating of Perceived Exertion (RPE) scale [31]. The entire rehabilitation program was personalized to align with each patient’s physical condition, rehabilitation goals, and subjective fatigue threshold.

### 2.5. Whole-Body Cryostimulation Procedure

The whole-body cryostimulation (WBC) procedure involved the exposure of participants’ entire bodies to extremely low temperatures (−110 °C) for a duration of two minutes, carried out inside a certified medical cryochamber cooled with liquid nitrogen (Artic, CryoScience, Rome, Italy), located at the IRCCS Istituto Auxologico Italiano, in Piancavallo, Italy. Prior to the start of the treatment cycle, each patient underwent an acclimatization session, consisting of a one-minute exposure at −110 °C to allow familiarization with the procedure. The WBC cycle included two sessions per day, from Monday to Friday, at 8:15 a.m. and at 12:00 p.m.

Before entering the cryochamber, patients were required to remove any metallic objects, including jewellery, as well as glasses and contact lenses, and were instructed to thoroughly dry their skin to prevent cold-induced skin injuries. Participants accessed the chamber wearing minimal clothing, such as shorts or sweatpants, a lightweight T-shirt or remaining shirtless (in the case of women, a sports bra was permitted). Particular attention was given to the protection of body extremities: mid-calf socks, clogs, gloves, head coverings, and earmuffs were provided to minimize the risk of frostbite. Additionally, surgical face masks were worn during the session to shield the oral and nasal mucosa from direct exposure to cold air and for hygienic purposes.

During the cryostimulation, participants were instructed to maintain normal breathing patterns and, if necessary, to shift their body weight or gently move their fingers to enhance comfort and tolerance to the cold exposure. Throughout each session, continuous supervision was ensured via both visual and verbal contact between staff and patients. Blood pressure (both systolic and diastolic) was systematically monitored immediately before and after each WBC session to ensure cardiovascular safety. All procedures were conducted and monitored by certified operators with specialized training in cryostimulation protocols (P.P. and F.V.).

### 2.6. Blood Sample Collection and Biochemical Analysis

Blood samples were collected from all participants through an indwelling cannula inserted at the cubital vein. Specifically, venipuncture was performed after overnight fasting, immediately before and within 15 min after the first whole-body cryostimulation session (Pre-T1 and Post-T1). Immediately following collection, the samples were centrifuged at 4200 rpm for 10 min at 4 °C, aliquoted and stored at −80 °C until analysis.

This same protocol was repeated before and after the last WBC treatment (Pre-T10 and Post-T10), ensuring identical conditions across all sampling points. Serum serotonin levels were determined using commercially available ELISA kits (IBL International, Hamburg, Germany). The intra- and inter- assay coefficients of variation were 3.8–6.6% and 6.7–17.3%, respectively. All samples were assayed in duplicate, in accordance with the manufacturer’s instructions.

Serum cortisol levels were measured using a chemiluminescent assay (Roche Diagnostics, Mannheim, Germany), considering a normal range of 4.82–19.5 μg/dL. All biochemical determinations were conducted according to validated procedures, ensuring assay reliability and reproducibility.

### 2.7. Psychological Assessment and General Well-Being

To evaluate the impact of whole-body cryostimulation (WBC) on psychological health and overall well-being, a comprehensive battery of validated self-report questionnaires was administered to all participants. The assessments were conducted immediately before the first WBC session and repeated immediately after the final session, in order to capture both baseline conditions and potential changes following the intervention.

Daytime sleepiness was measured using the Epworth Sleepiness Scale (ESS), a widely used tool that assesses the tendency to fall asleep during various daily situations [32]. This scale was selected due to the high prevalence of excessive daytime sleepiness in patients with Parkinson’s disease (PD), which often worsens quality of life and contributes to fatigue [33].

Fatigue levels were assessed with the Fatigue Severity Scale (FSS), which is specifically designed to quantify the severity and impact of fatigue on daily functioning [34]. Fatigue is one of the most common and disabling non-motor symptoms in PD, frequently reported by patients as a major limiting factor in their daily activities [35].

Anxiety symptoms were evaluated using the State-Trait Anxiety Inventory (STAI) forms I and II, which distinguish between transient anxiety related to specific situations (state anxiety, STAI-I) and more stable, long-term anxiety tendencies (trait anxiety, STAI-II) [36]. Given the high prevalence of anxiety in PD and its significant effect on mood and functioning, this instrument was chosen to provide a detailed profile of anxiety changes during the intervention [37].

Depressive symptoms were measured through the Beck Depression Inventory (BDI), a well-established questionnaire that assesses the severity of depression [38]. Depression is a frequent comorbidity in PD, often underdiagnosed and undertreated, with substantial repercussions on quality of life and disease management [39].

Sleep quality and the presence of insomnia symptoms were assessed using the Sleep Condition Indicator (SCI), a validated measure designed to screen for insomnia and related sleep disturbances [40]. Sleep disorders are highly prevalent in PD, contributing to daytime dysfunction and exacerbating other non-motor symptoms such as mood disturbances and fatigue [41].

This combination of questionnaires was selected to comprehensively assess the key dimensions of psychological well-being and non-motor symptom burden commonly affected in PD, enabling a multidimensional evaluation of WBC’s effects on the overall health status of the participants.

### 2.8. Feasibility

Adherence to the WBC intervention was carefully monitored throughout the study period. Completion rates for both the biochemical assessments and the self-reported questionnaires were recorded at baseline and following the intervention. In addition, the occurrence of any adverse events was systematically tracked during the entire course of the study. No adverse reactions related to the WBC sessions or the overall rehabilitation program were observed.

Any participant withdrawals that occurred during the study were attributed to personal or unrelated reasons and were not linked to poor tolerance of either the cryostimulation protocol or the multidisciplinary rehabilitation program. On the contrary, the WBC treatment was well tolerated by all individuals who completed the intervention, with no reports of significant discomfort or safety concerns.

### 2.9. Study Outcomes

The primary outcomes of the study focused on circulating biochemical markers associated with stress response and overall well-being. Specifically, serum concentrations of cortisol and serotonin were measured at baseline and after the intervention, as these hormones are closely related to stress regulation, mood, and general health status.

Secondary outcomes consisted of validated self-reported questionnaires assessing psychological health, fatigue, sleep quality, and anxiety. These included the Beck Depression Inventory (BDI), State-Trait Anxiety Inventory (STAI forms I and II), Epworth Sleepiness Scale, Fatigue Severity Scale (FSS), and Sleep Condition Indicator (SCI), administered before and after the WBC cycle.

A comprehensive schematic representation of the study design and outcome assessments is provided in Figure 1.

### 2.10. Statistical Analysis

Statistical analyses were performed using Jamovi (version 2.7) [42]. Continuous variables were expressed as mean, median, standard deviation (SD), and standard error (SE). To assess the distributional assumptions, the Shapiro–Wilk test was applied to all pre-post difference scores. When normality was confirmed (*p* > 0.05), paired-sample Student’s *t*-tests were conducted to compare pre- and post- intervention values for each biomarker and questionnaire. A significance level of *p* < 0.05 was set for all statistical tests.

The analysis focused on changes in serum levels of cortisol and serotonin, as well as variations in psychometric scores (BDI, STAI-Y1 and Y2, Epworth Sleepiness Scale, FSS, and SCI), following the WBC intervention. When normality assumptions were violated, test results were still interpreted cautiously due to the small sample size and exploratory nature of the study. Significant differences were identified primarily in cortisol, serotonin, noradrenaline, and selected psychometric outcomes. Effect sizes were calculated for all paired comparisons using Cohen’s *dz* and were interpreted alongside *p*-values to provide additional information on the magnitude and potential clinical relevance of the observed effects, while acknowledging the exploratory nature and limited sample size of the study.

## 3. Results

### 3.1. Demographic and Descriptive Statistics

A total of 14 adult inpatients (9 males and 5 females, mean age 64.6 ± 9.1 years; mean disease duration 5.4 ± 2.3 years) with Parkinson’s disease agreed to participate in this study. Table 1 details the descriptive statistics before and after the first WBC session (Pre-T1 and Post-T1) and before and after the last (Pre-T10 and Post-T10).

### 3.2. Biochemical Markers of Stress and Well-Being

#### 3.2.1. Cortisol

Cortisol levels were measured in serum samples collected before and after both the first (T1) and the last (T10) whole-body cryostimulation (WBC) sessions. No statistically significant difference was observed immediately after the first session (Pre-T1 vs. Post-T1; t(13) = 1.256, *p* = 0.231), which was associated with a small effect size (Cohen’s *dz* = 0.336).

In contrast, a significant reduction in cortisol concentration was detected at the end of the full 10-session WBC cycle. This decrease was evident both when comparing pre- and post-T10 values (t(13) = 2.227, *p* = 0.044), with a moderate effect size (Cohen’s *dz* = 0.595), and when comparing baseline values before the first session with post-T10 measurements (t(13) = 3.039, *p* = 0.009), which showed a large effect size (Cohen’s *dz* = 0.812).

**Figure 2:**
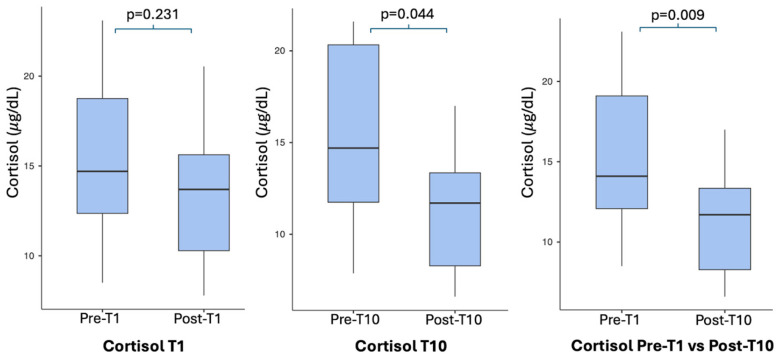
Changes in serum cortisol concentrations.

While a single WBC exposure shows a tendency to reduce cortisol level, not statistically significant, repeated sessions were associated with a progressively stronger and clinically meaningful reduction in stress-related hormonal responses. Table 2 and Figure 2 illustrate the paired comparisons and the temporal evolution of serum cortisol levels across the intervention period.

#### 3.2.2. Serotonin

Serum serotonin levels showed a statistically significant increase following both the first and the last whole-body cryostimulation (WBC) sessions. Specifically, a significant increase was observed immediately after the first session (Pre-T1 vs. Post-T1; t(13) = −2.274, *p* = 0.041), which was associated with a moderate effect size (Cohen’s *dz* = 0.608). A comparable increase was also detected after the completion of the 10-session intervention when comparing pre- and post-T10 values (t(13) = −2.184, *p* = 0.048), with a moderate effect size (Cohen’s *dz* = 0.584).

When comparing baseline values (Pre-T1) with measurements obtained at the end of the protocol (Post-T10), serum serotonin levels showed a trend toward statistical significance (t(13) = −2.006, *p* = 0.066), accompanied by a moderate effect size (Cohen’s *dz* = 0.536).

**Figure 3:**
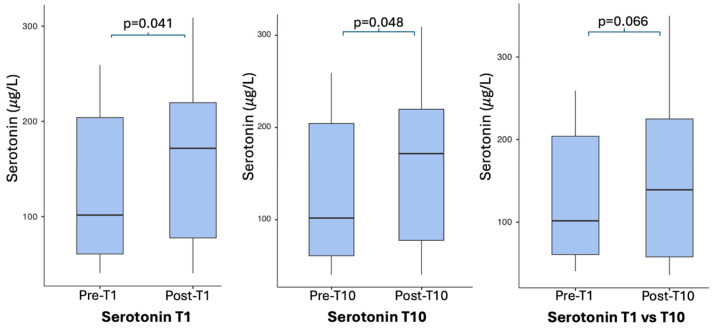
Changes in serum serotonin concentrations.

Overall, the effect size analysis indicates that WBC was associated with consistent, moderate increases in circulating serotonin levels across the intervention period, even when statistical significance was not reached for the baseline-to-end comparison. Table 3 and Figure 3 summarize the paired comparisons and visually illustrate the temporal changes in serum serotonin concentrations.

### 3.3. Patient-Reported Questionnaire Results

The administration of validated questionnaires before and after the 10-session whole-body cryostimulation (WBC) intervention revealed significant improvements in several patient-reported outcomes related to psychological health and general well-being. Depressive symptoms, assessed by the Beck Depression Inventory (BDI), showed a significant reduction following the intervention (t(10) = 3.236, *p* = 0.009), associated with a large effect size (Cohen’s *dz* = 0.973).

Similarly, anxiety levels significantly improved after WBC exposure. State anxiety (STAI-Y1) showed a robust decrease (t(9) = 4.60, *p* = 0.001), with a very large effect size (Cohen’s *dz* = 1.454), while trait anxiety (STAI-Y2) also significantly decreased (t(10) = 2.56, *p* = 0.028), with a moderate-to-large effect size (Cohen’s *dz* = 0.772). These findings indicate a consistent anxiolytic effect of the intervention across both state- and trait-related dimensions.

Subjective daytime sleepiness, assessed by the Epworth Sleepiness Scale, significantly improved after the intervention (t(10) = 4.57, *p* = 0.001), showing a very large effect size (Cohen’s *dz* = 1.379). Perceived fatigue, measured by the Fatigue Severity Scale (FSS), also showed a statistically significant reduction (t(10) = 2.25, *p* = 0.048), with a moderate effect size (Cohen’s *dz* = 0.679).

Conversely, no significant changes were observed in sleep quality as assessed by the Sleep Condition Indicator (SCI) (t(10) = 0.00, *p* = 1.000), with a null effect size (Cohen’s *dz* = 0.000), indicating the absence of measurable effects of WBC on insomnia-related symptoms in this cohort.

**Figure 4:**
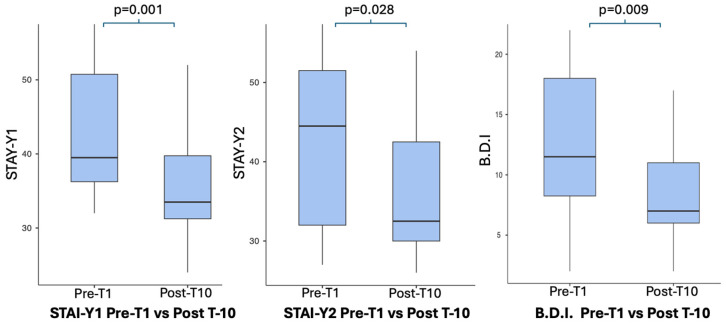
Changes in psychological outcomes (STAI-Y1 and Y2, BD).

**Figure 5:**
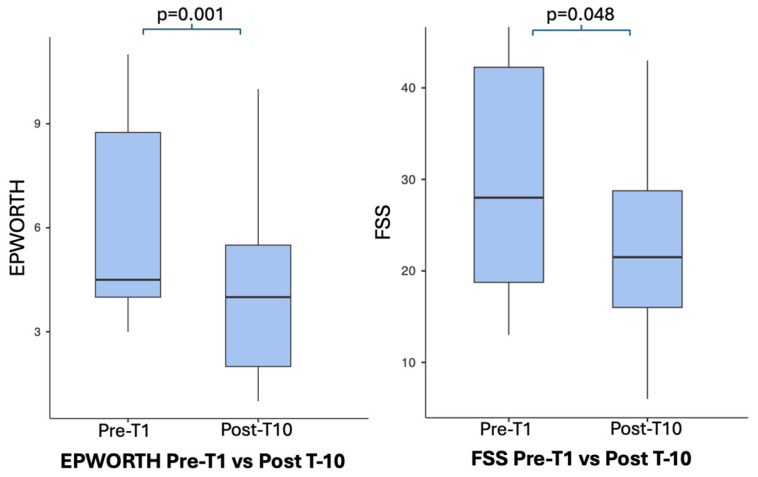
Changes in psychological outcomes (EPWORTH and FSS).

Psychological scale results are summarized in Table 4 and visually illustrated in Figure 4 (STAI-Y1, STAI-Y2, and BDI) and Figure 5 (Epworth and FSS).

## 4. Discussion

This pilot study investigated the impact of integrating whole-body cryostimulation (WBC) into a multidisciplinary rehabilitation program on selected stress and well-being related biomarkers (cortisol and serotonin), as well as on psychological and Non-Motor Symptoms (NMS) outcomes in patients with Parkinson’s disease (PD). This analysis was conducted on the same cohort of PD patients previously investigated for cardiac autonomic responses to WBC exposure, but here we focused on a different dimension: neuroendocrine and psychological parameters [20].

To the best of our knowledge, this is the first study investigating the effects of a multidisciplinary rehabilitation program that includes WBC on stress- and well-being–related neuroendocrine markers, as well as on NMS in patients with Parkinson’s disease.

Although cortisol is widely recognized as a biomarker of psychological and physiological stress [43], the evidence regarding its modulation by whole-body cryostimulation (WBC) remains inconsistent. Some investigations have reported significant post-session increases in cortisol levels following WBC, presumed to reflect an acute stress response to extreme cold stimuli [22]. Other studies have found no significant alterations in cortisol profiles after WBC exposure [21,44]. Moreover, in subjects undergoing regular cold exposure, including winter swimming or WBC, cortisol concentration decreased over time, likely reflecting neuroendocrine adaptation and stress desensitization [45].

In the present study, serum cortisol levels did not show a statistically significant change following the first WBC session (*p* = 0.231), and this acute response was associated with a small effect size, suggesting a limited immediate impact of a single exposure. In contrast, a significant acute reduction in cortisol was observed after the final WBC session (*p* = 0.044), accompanied by a moderate effect size, indicating a more pronounced stress-related hormonal response after repeated exposures. Moreover, when comparing baseline cortisol levels prior to the first session with those measured after the last treatment, a statistically significant decrease was detected (*p* = 0.009), with a large effect size, supporting the presence of a cumulative effect of repeated WBC sessions on hypothalamic–pituitary–adrenal (HPA) axis activity. Elevated cortisol levels appear to be associated with anxiety, sleep disturbances, depressive symptoms, and altered stress responsiveness, all of which represent prevalent and burdensome non-motor symptoms in Parkinson’s disease [46]. In this context, the progressive reduction in cortisol observed after repeated WBC exposure may reflect a shift towards a more regulated neuroendocrine stress profile, which may require days to stabilize. Although causal inferences cannot be drawn from this pilot design, the magnitude of the observed effect suggests that WBC may contribute to modulating stress-related neuroendocrine mechanisms in PD, where chronic HPA axis hyperactivity contributes to stress-related symptoms. By attenuating cortisol levels, WBC may help alleviate anxiety and depressive manifestations that profoundly impair quality of life in this population.

The increase in serotonin, a key neurotransmitter involved in mood regulation and fatigue resistance, further reinforces the potential of WBC in targeting non-motor symptoms in PD. Growing evidence from animal, biochemical, post-mortem and human in vivo studies has demonstrated loss of striatal and extra-striatal serotonin markers in the course of PD, indicating that the serotonergic system is affected by PD pathology [47,48]. Serotoninergic dysfunction is therefore emerging as a pivotal factor in Parkinson’s disease (PD), particularly in relation to non-motor symptoms [48]. In animal models, a selective loss of serotonergic fibers has been documented in key brain areas, including the hippocampus, potentially underpinning manifestations such as anxious depression and impaired neurogenesis in PD [49]. Additional evidence highlights that serotonergic degeneration may precede dopaminergic loss and contribute not only to mood-related symptoms but also to motor features like tremors [50].

Whole-body cryostimulation (WBC) has been shown to increase circulating serotonin levels through neuroendocrine mechanisms in healthy and chronic-pain populations. Barlowska-Trybułec et al. [24] reported increases in plasma serotonin following WBC combined with kinesitherapy in patients with osteoarthrosis of the lumbar spine. Translated to PD patients, enhancing serotonin acutely post-WBC and cumulatively over repeated sessions may help alleviate debilitating non-motor symptoms like depression, anxiety, fatigue and cognition [51], as well as some of the motor symptoms (tremors) [50]. By raising serotonin, WBC may improve these symptoms, offering a non-pharmacological, well-tolerated adjunctive treatment in PD rehabilitation pathways.

In the present study, serum serotonin levels showed a statistically significant increase following both the first WBC session (*p* = 0.041) and the completion of the 10-session protocol (*p* = 0.048). Both acute responses were associated with moderate effect sizes, indicating a consistent serotonergic response to cryostimulation even after a single exposure. When comparing baseline levels with those measured at the end of the intervention, a trend toward statistical significance was observed (*p* = 0.066), accompanied by a moderate effect size, suggesting that the magnitude of the effect remained clinically meaningful despite not reaching conventional statistical thresholds.

These findings are particularly noteworthy and warrant further validation through larger-scale, randomized controlled trials with robust methodological designs. Given the complex interplay between serotonergic dysfunction and the manifestation of non-motor symptoms in Parkinson’s disease, the observed increase in circulating serotonin following WBC represents a promising therapeutic signal. If replicated in broader cohorts, this effect could pave the way for the integration of WBC into multidisciplinary rehabilitation protocols aimed not only at alleviating depression, anxiety, and fatigue but also at enhancing overall well-being and health-related quality of life in PD patients. Such non-pharmacological approaches may be especially valuable in a population often burdened by polypharmacy and limited treatment options for NMS [52,53].

In addition to biochemical changes, the intervention was associated with significant improvements in several patient-reported outcomes related to psychological health and daytime functioning. Depressive symptoms, assessed by the Beck Depression Inventory (BDI), significantly decreased following the WBC program (*p* = 0.009), with a large effect size, indicating a clinically meaningful reduction in depressive burden. Similarly, anxiety levels improved substantially, with marked reductions observed in both state anxiety (STAI-Y1; *p* = 0.001) and trait anxiety (STAI-Y2; *p* = 0.028), accompanied by large and moderate-to-large effect sizes, respectively. These findings suggest a robust anxiolytic effect of the intervention.

Improvements were also observed in domains related to daytime sleepiness and fatigue. Subjective daytime sleepiness significantly decreased after the intervention (Epworth Sleepiness Scale; *p* = 0.001), showing a very large effect size, while perceived fatigue, measured by the Fatigue Severity Scale (FSS), also showed a statistically significant reduction (*p* = 0.048), with a moderate effect size. In contrast, no significant changes were detected in sleep quality as assessed by the Sleep Condition Indicator (SCI; *p* = 1.000), with a null effect size, indicating that WBC did not measurably influence insomnia-related symptoms in this cohort, possibly because sleep disorders are related to disregulation of several neural pathways and have a multifactorial origin.

Several previous studies have reported beneficial effects of WBC on mood, anxiety, fatigue, and sleep-related parameters in both healthy individuals and clinical populations [25,54,55,56]. To date, only one study has explored the effects of WBC in patients with Parkinson’s Disease, reporting improvements in cardiac autonomic balance and confirming safety in patients with this condition [20].

The present findings align with this growing body of evidence, further supporting the hypothesis that WBC may serve as a valuable add-on to rehabilitation interventions to improve psychosocial well-being in patients with Parkinson’s disease. By confirming positive trends in these domains, our study reinforces the potential role of WBC as a non-pharmacological strategy for addressing debilitating non-motor symptoms and enhancing overall quality of life in PD patients.

Notably, the absence of adverse events or significant blood pressure fluctuations throughout the protocol confirms the safety and tolerability of WBC in this vulnerable patient population. This is particularly relevant given the high prevalence of autonomic dysfunction and orthostatic hypotension in PD, where interventions that might destabilize cardiovascular function must be approached cautiously.

Taken together, these findings suggest that WBC may offer a dual benefit in PD rehabilitation: improving autonomic regulation, as previously demonstrated, and concurrently enhancing neuroendocrine balance and psychological well-being. This integrative effect positions WBC as a promising “rehabilitation enhancer” for PD, capable of addressing both physiological and psychological aspects of the disease.

The multifaceted nature of Parkinson’s disease underscores the need for a holistic approach that complements conventional pharmacological and surgical therapies. Integrating multidisciplinary rehabilitation strategies such as physiotherapy, exercise, and adjunctive interventions like WBC may more effectively address the interplay between motor and non-motor symptoms, ultimately enhancing patients’ overall well-being and quality of life [57].

However, several limitations must be acknowledged. The small sample size, the absence of a control group, the non-randomized design, and the relatively short duration of the intervention limit the generalizability of our findings. In addition, the presence of several confounding components inherent to the multidisciplinary rehabilitation program, including physiotherapy and physical exercise, prevents the isolation of the specific contribution of whole-body cryostimulation. Moreover, variability in patient age and disease duration may have influenced individual responses, although the sample was relatively homogeneous in terms of disease severity and clinical subtype. Finally, the lack of a follow-up assessment does not allow conclusions to be drawn regarding the persistence of the observed effects over time. As a pilot study, these limitations were anticipated; nevertheless, the consistency of the results across biological and psychological domains supports the rationale for future larger-scale investigations. In conclusion, this pilot study provides preliminary evidence that a rehabilitation program including whole-body cryostimulation may improve stress-related biomarkers, neurotransmitter levels, and psychological well-being in patients with Parkinson’s disease. These results, in conjunction with our prior findings on autonomic modulation, suggest that WBC could serve as a valuable complementary therapy in the multidisciplinary management of PD. Future randomized controlled trials with larger cohorts and longer follow-up periods will be essential to confirm these initial findings and clarify the long-term clinical benefits of WBC in this population.

## 5. Conclusions

The findings of this pilot study suggest that a 10-session whole-body cryostimulation (WBC) protocol added to a comprehensive multidisciplinary rehabilitation program may beneficially influence stress-related hormonal responses and psychological well-being in patients with Parkinson’s disease. Specifically, we observed reductions in cortisol, increases in serotonin, and significant improvements in mood, anxiety, sleep quality, and fatigue levels.

These results, obtained from the same patient cohort previously analyzed for autonomic responses, highlight the potential of WBC not only in modulating cardiac autonomic function but also in enhancing neuroendocrine balance and alleviating non-motor symptoms in PD. Furthermore, the intervention proved to be safe and well-tolerated, without adverse events or hemodynamic instability, confirming its feasibility in this clinical population.

Taken together, these findings suggest that WBC may represent a promising complementary strategy in the multidisciplinary management of Parkinson’s disease, particularly for improving general health status and psychological outcomes. However, further randomized controlled studies with larger sample sizes and extended follow-up periods are necessary to confirm these preliminary results and better define the therapeutic role of WBC in PD rehabilitation.

## Figures and Tables

**Table 1:** Descriptive statistics before and after the first (T1) and the last (T10) WBC session.

Outcome	N	Mean	Median	SD	SE
Cortisol Pre-T1 (μg/dL)	14	15.42	14.70	4.64	1.24
Cortisol Post-T1 (μg/dL)	14	13.7	13.69	4.00	1.06
Cortisol Pre-T10 (μg/dL)	14	15.0	14.70	4.99	1.33
Cortisol Post-T10 (μg/dL)	14	11.32	11.70	3.42	0.91
Serotonin Pre-T1 (μg/L)	14	128.07	101.60	78.17	20.89
Serotonin Post-T1(μg/L)	14	155.34	171.65	88.97	23.78
Serotonin Pre-T10 (μg/L)	14	115.00	103.17	64.47	17.23
Serotonin Post-T10 (μg/L)	14	154.88	139.10	100.65	26.90
BDI Pre-T1 (0–63)	11	12.09	11	6.98	2.10
BDI Post-T10 (0–63)	11	8.00	7	4.75	1.43
STAI 1 Pre-T1 (20–80)	10	43.00	39.5	8.93	2.82
STAI 1 Post-T10 (20–80)	10	36.60	33.5	9.07	2.87
STAI Y2 Pre-T1 (20–80)	11	41.91	40	11.34	3.42
STAI Y2 Post-T10 (20–80)	11	35.64	32	9.62	2.90
SCI Pre-T1 (0–32)	11	20.36	20	6.73	2.03
SCI Post-T10 (0–32)	11	20.36	19	7.00	2.11
Epworth Pre-T1 (0–24)	11	6.82	5	4.12	1.24
Epworth Post-T10 (0–24)	11	4.18	4	2.64	0.79
FSS Pre-T1 (9–63)	11	29.00	27	12.43	3.75
FSS Post-T10 (9–63)	11	24.00	22	13.80	4.16

Table 1 abbreviation list: WBC: Whole-body cryostimulation; T1: First WBC session; T10: Last WBC session; Pre: Before session; Post: After session; BDI: Beck Depression Inventory; STAI Y1: State-Trait Anxiety Inventory, Form Y-1 (State Anxiety); STAI Y2: State-Trait Anxiety Inventory, Form Y-2 (Trait Anxiety); SCI: Sleep Condition Indicator; FSS: Fatigue Severity Scale.

**Table 2:** Paired *t*-test comparisons of serum cortisol levels before and after the first (T1) and the last (T10) WBC session.

		Test	*t*-Value	df	*p*-Value	Effect Size (Cohen’s *dz*)
Cortisol Pre-T1	Cortisol Post-T1	Student’s t	1.256	13	0.231	0.336
Cortisol Pre-T10	Cortisol Post-T10	Student’s t	2.227	13	**0.044**	0.595
Cortisol Pre-T1	Cortisol Post-T10	Student’s t	3.039	13	**0.009**	0.812

Bold: significant difference values.

**Table 3:** Paired *t*-test comparisons of serum serotonin levels before and after the first (T1) and the last (T10) WBC session.

		Test	*t*-Value	df	*p*-Value	Effect Size (Cohen’s *dz*)
Serotonin Pre-T1	Serotonin Post-T1	Student’s t	−2.274	13	**0.041**	−0.608
Serotonin Pre-T10	Serotonin Post-T10	Student’s t	−2.184	13	**0.048**	−0.584
Serotonin Pre-T1	Serotonin Post-T10	Student’s t	−2.006	13	0.066	−0.536

Bold: significant difference values.

**Table 4:** Paired *t*-test comparisons of psychological scales and questionnaires before and after the first (T1) and the last (T10) WBC session.

		Test	*t*-Value	df	*p*-Value	Effect Size (Cohen’s *dz*)
BDI Pre-T1	BDI Post-T10	Student’s t	3.236	10	**0.009**	0.973
STAI-Y1 Pre-T1	STAI-Y1 Post-T10	Student’s t	4.60	9.00	**0.001**	1.454
STAI-Y2 Pre-T1	STAI-Y2 Post-T10	Student’s t	2.56	10.00	**0.028**	0.772
SCI Pre-T1	SCI Post-T10	Student’s t	0.00	10.00	1.000	0.000
Epworth Pre-T1	Epworth Post-T10	Student’s t	4.57	10.00	**0.001**	1.379
FSS Pre-T1	FSS Post-T10	Student’s t	2.25	10.00	**0.048**	0.679

Bold: significant difference values.

## Data Availability

Data are available on request in the Zenodo repository (10.5281/zenodo.17615794) due to restrictions, e.g., privacy or ethical.
